# Elevated expansion of follicular helper T cells in peripheral blood from children with acute measles infection

**DOI:** 10.1186/s12865-020-00379-4

**Published:** 2020-09-01

**Authors:** Weiyun Shen, Hongzhou Ye, Xilin Zhang, Lixia Huo, Jingli Shen, Li Zhu, Xiang Wang, Dawei Cui

**Affiliations:** 1 Key Laboratory for Translational Medicine, First Affiliated Hospital, Huzhou Teachers College, the First People’s Hospital of Huzhou, Huzhou, 313000 China; 2 Department of Paediatrics, First Affiliated Hospital, Huzhou Teachers College, the First People’s Hospital of Huzhou, Huzhou, 313000 China; 3 Department of Blood Transfusion, the First Affiliated Hospital, Zhejiang University School of Medicine, Hangzhou, 310003 Zhejiang China

**Keywords:** Measles virus, Follicular helper T cells, Bcl-6, IL-21, Neutralizing antibody

## Abstract

**Background:**

Measles outbreaks have threatened the global elimination and eradication of measles in recent years. Measles virus (MeV)-specific antibodies are successful in clearing MeV infection. Follicular helper T (Tfh) cells play a crucial role in promoting antibody production. This study investigated the potential role of Tfh cells in peripheral blood mononuclear cells (PBMCs) from children with acute MeV infection.

**Results:**

The frequencies of CXCR5^+^CD4^+^ Tfh, ICOS^high^ Tfh, and PD-1^high^ Tfh cells in PBMCs and levels of IL-6 and IL-21 in plasma were significantly elevated in patients with acute MeV infection. Moreover, a positive correlation was discovered among the frequency of ICOS^high^ Tfh cells, plasma levels of IL-21 and optical density (*OD*) values of MeV-specific IgM antibodies in the patients with acute MeV infection. However, elevated plasma MeV-specific NAb titres were not associated with the frequency of Tfh, ICOS^high^ Tfh, or PD-1^high^ Tfh cells in the patients with acute MeV infection.

**Conclusion:**

These results suggest that an elevated Tfh cell frequency and associated molecules possibly play a key role in children with acute MeV infection, which contributes to the prevention and treatment of MeV infection in children.

## Background

Measles, which is caused by measles virus (MeV), is a highly contagious disease characterized by fever, maculopapular rash, conjunctivitis, cough and Koplik spots, and other complications include pneumonia, encephalitis, and death [1, 2]. The measles vaccine is a highly safe and effective vaccine for controlling and interrupting MeV infection; however, cases of measles have risen to high levels globally in recent years [3–7]. An estimated 110,000 deaths were reported in 2017, most of which were children under 5 years of age [8, 9]. Measle cases rose by 300% in the first three months of 2019 in comparison with the same period in 2018 [10]. The elimination and eradication of measles remains a serious and emergent problem worldwide.

Evidence suggests that MeV-specific IgM/IgG antibody titres in serum are unexpectedly low among cohorts who had high coverage of two-dose vaccination, and low levels of MeV-specific antibodies are closely associated with outbreaks of measles infection; conversely, high levels of MeV-specific antibodies are required to achieve elimination and eradication of MeV infection [4, 11–13]. MeV-specific antibodies are produced by mature B cells that are helped by Th2 cells characterized by the cytokines interleukin (IL)-4, IL-10 and IL-13 [2, 14]. Recent studies indicate that follicular helper T (Tfh) cells play a crucial role in promoting responses in the germinal centre (GC) by B cells that can secrete specific antibodies, and Tfh cells are characterized by expression of chemokine (CXC motif) receptor 5 (CXCR5), programmed death-1 (PD-1), and inducible costimulator (ICOS); high secretion of IL-21; and activity of B-cell lymphoma-6 (Bcl-6) as a critical transcription factor [15–18]. Peripheral ICOS^high^CXCR5^+^CD4^+^ Tfh (ICOS^high^ Tfh) and/or PD-1^high^ Tfh cells have been defined as circulating Tfh cells in the peripheral blood, and circulating Tfh cells are closely related to autoantibody levels in autoimmune diseases [19]. To date, there have been few studies on Tfh cells in the peripheral blood of children with acute MeV infection.

The present research explores the role of Tfh cells, associated molecules and plasma MeV-specific antibodies in peripheral blood from children with acute MeV infection. These results indicate that an elevated frequency of Tfh cells plays a crucial role in children with MeV infection, which provides new insights into the prevention and treatment of MeV infection.

## Results

### Demographics of the MeV patients

Among 42 patients, 9 (21.4%) were negative, 33 (78.6%) were positive for MeV-specific IgM antibodies, and 26 (61.9%) were positive for MeV-specific IgG antibodies. Moreover, the results showed that 8 cases were associated with the Chinese vaccine strain Shanghai-191 (GenBank accession No. FJ416067) and 34 cases were positive for wild-type MeV infection. However, 18 (90%) children were positive for MeV-specific IgG antibodies in the HC group, and the results indicated that the HCs were negative for MeV-specific IgM antibodies and MeV RNA.

The mean age of the patients, including 19 females and 23 males, was 3.6 years, and the mean age of the HCs, including 9 females and 11 males, was 3.5 years. Thirty-nine patients and 20 HC children had two doses of the measles vaccine, and only 3 patients were unvaccinated. The patients with MeV infection had fever (100%), maculopapular rash (100%), cough (83.3%) and conjunctivitis (54.8%). The results are shown in Table 1.

**Table 1:** Clinical characteristics of children with acute MeV infection

Clinical characteristics	MeV	HC
Number	42	20
Age (years)	3.6 ± 1.2	3.5 ± 1.1
Sex (M/F)	23/19	11/9
Maculopapular rash	42 (100%)	N
Fever	42 (100%)	N
Cough	35 (83.3%)	N
Conjunctivitis	23 (54.8%)	N
Koplik spots	19 (45.2%)	N
Chronic bronchitis	10 (23.8%)	N
Viral RNA of MeV	Positive	Negative
IgM (negative/positive)	9/33	0/0
IgG (negative/positive)	16/26	2/18
Time of the last booster vaccination	18 ~ 24 Mon^a^	18 ~ 24 Mon

Note: *M/F* Male/female, *HC* Healthy controls, *MeV* Measles virus, *N* Normal, *Mon* Month. ^a^, 3 of 42 cases were unvaccinated in the study. In China, two doses of routine vaccination of measles were performed, the age of children was 8 months for the first dose, and the last booster vaccination was 18 ~ 24 months

### Elevated frequencies of Tfh cells in patients with acute MeV infection

**Fig. 1:**
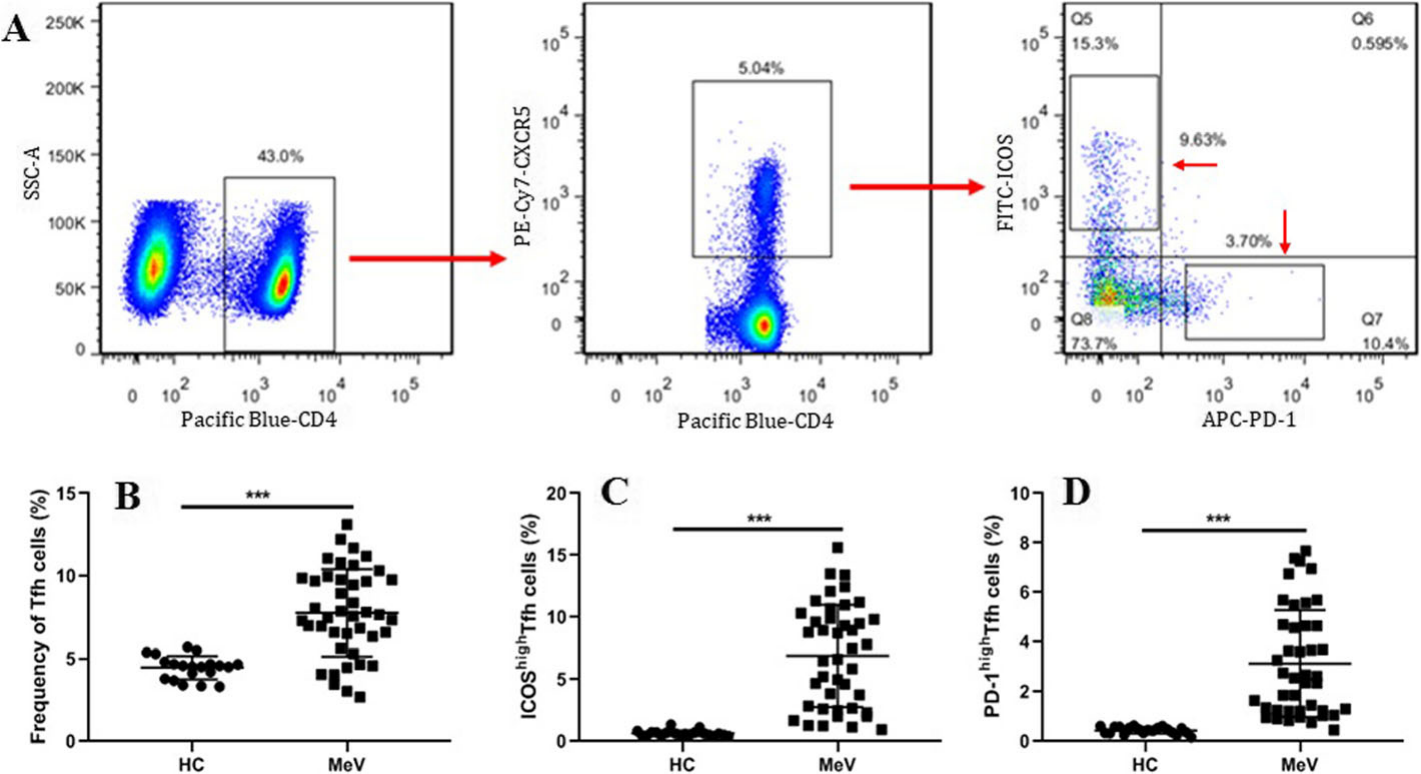
Elevated frequency of CXCR5^+^CD4^+^ Tfh cells in the peripheral blood of patients with acute MeV infection. **a** The frequency of CXCR5^+^CD4^+^ Tfh cells with an ICOS^high^ and PD-1^high^ phenotype in PBMCs from the patients with MeV infection was detected using flow cytometry; **b** CXCR5^+^CD4^+^ Tfh cells in the patients with MeV infection; **c** ICOS^high^CXCR5^+^CD4^+^ Tfh cells in the patients with MeV infection; **d** PD-1^high^CXCR5^+^ Tfh cells in the patients with MeV infection. The horizontal lines represent the means. ***, *P* < 0.0001

To explore the potential role of Tfh cells in the peripheral blood of the patients with acute-phase MeV infection, the frequencies of CD4^+^CXCR5^+^ Tfh, ICOS^high^ Tfh and PD-1^high^ Tfh cells in PBMCs were detected by flow cytometry (Fig. 1a). The results indicated that the frequency of CD4^+^CXCR5^+^ Tfh cells was surprisingly increased in the patients with acute MeV infection in comparison with the HCs (Fig. 1b). In addition, the frequencies of ICOS^high^ Tfh cells and PD-1^high^ Tfh cells were obviously increased in the patients with MeV infection in comparison with the HCs (Fig. 1c, d).

### Correlation of plasma MeV-specific antibody levels and Tfh cell frequencies

**Fig. 2:**
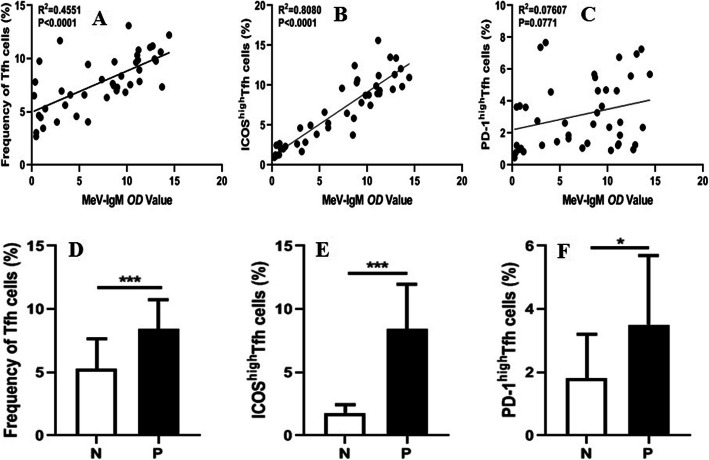
Correlation of plasma MeV-specific IgM *OD* values and circulating Tfh cells in MeV-infected patients. **a** Relationship of plasma MeV-specific IgM *OD* values and the percentage of CXCR5^+^CD4^+^ Tfh cells; **b** Relationship of plasma MeV-specific IgM *OD* values and the percentage of ICOS^high^ Tfh cells; **c** Relationship of plasma MeV-specific IgM *OD* values and the percentage of PD-1^high^ Tfh cells; **d** Different frequencies of CXCR5^+^CD4^+^ Tfh cells in the patients with negative (N) and positive (P) plasma MeV-specific IgM antibody results; **e** Different frequencies of ICOS^high^ Tfh cells in the patients with negative (N) and positive (P) plasma MeV-specific IgM antibody results; **f** Different frequencies of PD-1^high^ Tfh cells in the patients with negative (N) and positive (P) plasma MeV-specific IgM antibody results. ***, *P* < 0.0001; *, *P* < 0.05

The frequencies of CXCR5^+^CD4^+^ Tfh and ICOS^high^ Tfh cells but not that of PD-1^high^ Tfh cells were positively correlated with the optical density (*OD*) values of plasma MeV-specific IgM antibodies from the patients during the acute phase of MeV infection (Fig. 2a, c). Interestingly, the frequencies of Tfh cells, ICOS^high^ Tfh cells and PD-1^high^ Tfh cells were significantly increased in the patients positive for MeV-specific IgM antibodies compared to those lacking specific IgM antibodies (Fig. 2d-f). However, the frequencies of Tfh, ICOS^high^ Tfh and PD-1^high^ Tfh cells were not correlated with the *OD* values of plasma MeV-specific IgG antibody levels (Fig. S1). Additionally, measles neutralizing antibody (NAb) titres were obviously lower in the vaccine strain (Shanghai-191) group than in the wild-type measles group, and the MeV-NAb titres were not also correlated with the frequency of Tfh, ICOS^high^ Tfh, or PD-1^high^ Tfh cells (Fig. S2).

### Elevated plasma IL-21 levels in patients with acute MeV infection

**Fig. 3:**
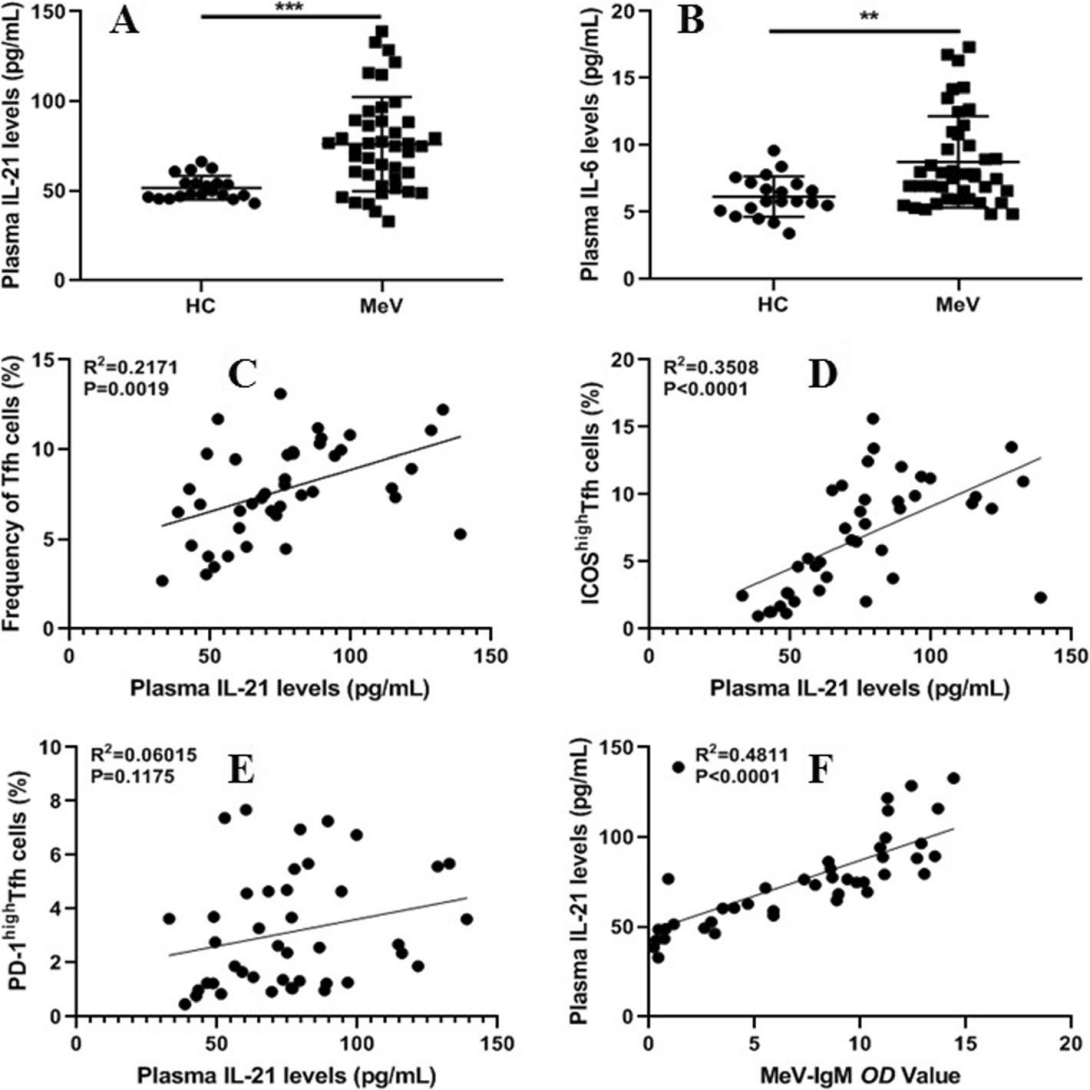
Correlations of cytokine levels and Tfh cells in the patients with MeV infection. **a**, **b** Levels of plasma IL-21 and IL-6 in the patients with MeV infection; **c**, **d**, **e** Relationships of plasma IL-21 concentrations with the frequencies of Tfh, ICOS^high^ Tfh and PD-1^high^ Tfh cells in MeV-infected patients, respectively; **f** Relationship of plasma IL-21 levels and the OD values of plasma MeV-specific IgM antibodies. ***, *P* < 0.0001; **, *P* < 0.01

To investigate the role of Tfh cell-associated cytokines in the plasma, the concentrations of IL-21 and IL-6 in the plasma of the patients with acute-phase MeV infection and that of the children in the HC group were tested by ELISA. The concentrations of IL-21 and IL-6 in the plasma were remarkably elevated in the patients with acute MeV infection compared to the HCs (Fig. 3a, b). Moreover, plasma IL-21 concentrations were positively related to the frequencies of Tfh cells, ICOS^high^ Tfh cells and the *OD* values of plasma MeV-specific IgM antibodies in the patients with acute-phase MeV infection (Fig. 3c, d, f); however, plasma IL-21 concentrations were not associated with the *OD* values of plasma MeV-specific IgG antibodies (data not shown). However, there was no correlation between the PD-1^high^ Tfh cell frequency and plasma IL-21 concentration in these patients (Fig. 3e). Additionally, plasma IL-6 levels were not significantly related to the frequency of Tfh cells, ICOS^high^ Tfh cells, or PD-1^high^ Tfh cells or to the *OD* values of plasma MeV-specific IgM antibodies in the patients with acute-phase MeV infection (data not shown).

### mRNA expression of IL-21, IL-6 and Bcl-6 in patients with acute MeV infection

**Fig. 4:**
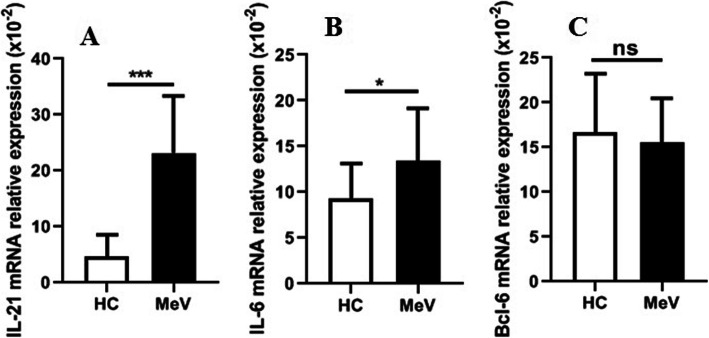
The relative mRNA expression levels of IL-21, IL-6 and Bcl-6 in PBMCs from MeV patients (*n* = 20) and HCs (*n* = 12). **a** Relative mRNA expression level of IL-21; b Relative mRNA expression level of IL-6; **c** Relative mRNA expression level of Bcl-6. ***, *P* < 0.0001; **, *P* < 0.01; ns, no significance

To further investigate the roles of genes encoding Tfh cell-associated molecules, the mRNA expression levels of IL-21, IL-6 and Bcl-6 in PBMCs isolated from the patients during the acute phase of MeV infection and HCs were assessed. The results suggested that the relative mRNA expression levels of IL-21 and IL-6 were remarkably increased in the PBMCs from the patients with acute MeV infection in comparison with those from the HCs, but Bcl-6 mRNA expression was not significantly different between the patients and HCs (Fig. 4a-c).

## Discussion

Measles vaccination is an effective measure to prevent and control MeV infection, although the variation in MeV and low levels of MeV-specific antibodies are important factors for the epidemic of MeV infection [2, 12–14, 20]. In this study, there were 33 (78.6%) positive patients with MeV-specific IgM antibodies, and 26 (61.9%) patients were detected to have MeV-specific IgG antibodies among 42 patients with MeV infection. Additionally, PCR results showed that all patients were positive for MeV infection, including 8 with vaccine-associated measles and 34 with wild-type measles. Interestingly, 18 (90%) children were positive for MeV-specific IgG antibodies. These results indicated that a low positive rate for MeV-specific IgG antibodies and wild-type MeV were mainly responsible for MeV infection. Therefore, a high level of MeV-specific neutralizing antibodies, surveillance of variation in MeV and design of novel MeV vaccines are required for elimination of MeV infection.

Early studies showed that Tfh cells and associated molecules facilitate the production of virus-specific antibodies to neutralize the virus by generating long-lived memory B cells and plasma cells [16, 21, 22]. Naive CD4^+^T cells are clearly polarized to Tfh cell fates with high Bcl-6 and CXCR5 expression after lymphocytic choriomeningitis virus (LCMV) infection or influenza virus infection, which is important for viral clearance; however, Tfh cells are virus reservoirs in patients infected with human immunodeficiency virus (HIV) or individuals infected with simian immunodeficiency virus (SIV) [16, 21–24]. During LCMV, HIV, or influenza virus infection, ICOS and PD-1 expression on Tfh cells is crucial for GC formation and humoural response induction [21–23, 25]. However, limited information is available about Tfh cells in MeV infection. In this study, the frequencies of CD4^+^CXCR5^+^ Tfh, ICOS^high^ Tfh and PD-1^high^ Tfh cells in peripheral blood were significantly increased in children with acute-phase MeV infection. In addition, the Tfh cell and ICOS^high^ Tfh cell frequencies were positively correlated with the *OD* values of plasma MeV-specific IgM antibodies. However, the frequencies of Tfh, ICOS^high^ Tfh and PD-1^high^ Tfh cells were not related to the *OD* values of plasma MeV-specific IgG antibody or measles neutralizing antibody (MeV-NAb) titres. These findings suggested that elevated percentages of Tfh cells, ICOS^high^ Tfh cells and PD-1^high^ Tfh cells might contribute to the induction of MeV-specific IgM antibody production during the acute phase of MeV infection. During the early phase of MeV infection, the MeV-specific IgG antibody and MeV-NAb titres were not rapidly induced by Tfh cells and their subsets. We will try to obtain follow-up serum samples to determine whether the presence of these circulating Tfh cells during the acute response correlates with better long-term antibody responses, including elevated MeV-specific IgG antibody and MeV-NAb levels, in the future, which is helpful for our research on the role of Tfh cells in MeV infection.

Recent research shows that IL-6 and IL-21 play a central role in regulating Tfh cell differentiation and function, including ICOS and PD-1 expression in Tfh cells [16, 26]. IL-6 is expressed by multiple cell types, including APCs and B cells, and IL-21 is predominantly produced by Tfh cells [16, 27]. IL-6 and IL-21 can drive Tfh cell differentiation by inducing expression of the transcription factor Bcl-6, and loss of IL-6 and IL-21 signalling significantly reduces the number of Tfh cells and attenuates viral clearance during chronic viral infections [27, 28]. Moreover, IL-21 is important for B cell differentiation and the antibody response, and loss of IL-21 signalling produces an obvious defect in the differentiation of long-lived plasma cells and in the maintenance of specific antibody levels during virus infection [23, 29, 30]. In this study, the expression of IL-6 in the plasma and PBMCs was significantly elevated in the patients with acute MeV infection, which was consistent with previous studies [31, 32]. Limited information is available about IL-21 levels in MeV infection. IL-21 levels were also increased in the plasma and PBMCs of the patients with acute MeV infection, and plasma IL-21 levels were closely correlated with the Tfh cell and ICOS^high^ Tfh cell frequencies and *OD* values of MeV-specific IgM antibodies but not with the *OD* values of MeV-specific IgG antibodies. These findings indicate that MeV infection can induce the expression of IL-6 and IL-21 molecules that contribute to an increase in the number of Tfh cells and humoural immunity during the acute phase of MeV infection. Bcl-6, which is expressed in multiple cell types, including Tfh cells and B cells, is one of the major transcription factors for Tfh cell differentiation and is regulated by various molecules, including CD40-CD40 ligand and ICOS-ICOS ligand [15–18, 33]. However, Bcl-6 mRNA expression was not significantly different between the patients with MeV infection and HCs. We concluded that in this study, we detected Bcl-6 mRNA expression in PBMCs but not in purified Tfh cells, which might indicate a generally negative effect on Bcl-6 mRNA expression.

Our study also had limitations. First, it included a relatively limited number of clinical cases and undetected titres of MeV-specific IgG antibodies and MeV-specific neutralizing antibodies; thus, it is impossible to extrapolate our results to the entire panel of MeV infections. Second, we studied the frequency of Tfh cells and some associated molecules in PBMCs rather than exploring the mechanism underlying Tfh cell differentiation and function.

## Conclusion

The present study showed that the frequencies of CXCR5^+^CD4^+^ Tfh cells, PD-1^high^ Tfh cells and ICOS^high^ Tfh cells; levels of Tfh cell-associated molecules, including IL-21, IL-6 and Bcl-6; and plasma levels of MeV-specific IgM antibodies were significantly changed in peripheral blood from children collected during the acute phase of MeV infection, which suggested that abnormally expanded CXCR5^+^CD4^+^ Tfh cells, ICOS^high^ Tfh cells and PD-1^high^ Tfh cells with increased IL-21 and IL-6 levels might promote the production of MeV-specific IgM antibodies in children with acute MeV infection. Therefore, these results implied that aberrantly increased Tfh cell levels and associated molecules might play a crucial role in children with acute MeV infection, which would have therapeutic implications for MeV infection.

## Methods

### Demographics of children

The acute cases of MeV infection were confirmed by characteristic clinical symptoms (such as maculopapular rash, high fever and/or cough, and conjunctivitis) and laboratory diagnosis, including at least one positive result for MeV-specific IgM antibodies and MeV RNA [20, 34]. MeV-specific IgM antibodies in plasma collected from the patients within 4 days of rash onset were detected with a commercially available enzyme-linked immune sorbent assay (ELISA) (Haitai Biotechnology, Zhuhai, China). MeV RNA was also tested with a quantitative real-time RT-PCR assay using throat, nasopharyngeal, urine and/or fluids of maculopapular rash samples [20]. Samples positive for MeV RNA were further amplified with an RT-PCR assay specific for the C terminus of the N gene of MeV [34]. The RT-PCR products purified with the Qiagen MinElute PCR Purification Kit (Qiagen, Hilden, Germany) were sequenced, and the sequences were analysed to identify the genotypes of MeV to discriminate the measles vaccine stain and wild-type virus by MEGA version 5.0 software (http://www.megasoftware.net). Forty-two children with acute MeV infection in the Department of Paediatrics and 20 healthy controls (HCs) matched by age and sex were recruited at the First People’s Hospital of Huzhou, Zhejiang Province, China.

### Measure of the measles neutralizing antibody (NAb) titre

Plasma was inactivated at 56 °C for 30 min and diluted from 1:2 to 1:1024. In a 96-well cell plate, 25 μL of diluted serum was mixed with 25 μL of MeV solution (100 TCID_50_) and neutralized for 1 h at 37 °C. Then, 100 μL of Vero/SLAM cells (2 × 10^6^ cells) were added to the plate with the serum-MeV mixture and cultured at 37 °C for 7 days. The cytopathic effect (CPE) was observed and recorded in the study. The NAb titre of the serum without any cytopathic effects was determined by calculating the reciprocal of the highest dilution of the serum without any CPEs [35].

### Isolation of peripheral blood mononuclear cells

Fresh peripheral blood specimens were collected from the HCs and patients with acute MeV infection. Isolation of peripheral blood mononuclear cells (PBMCs) was performed utilizing a Ficoll-Hypaque solution (CL5020, Netherlands) following the manufacturer’s protocol. Next, the PBMCs were carefully washed twice with phosphate-buffered saline (PBS) and transferred into sterile tubes.

### Flow cytometry

Single-cell suspensions were obtained, and surface markers were stained according to the manufacturers’ protocols for relevant fluorochrome-conjugated anti-human antibodies: anti-CD4-Pacific Blue, anti-CXCR5 (CD185)-PE-Cy7, anti-ICOS (CD278)-FITC, anti-PD-1 (CD279)-APC, and matched isotype controls (BioLegend, San Diego, CA). The samples were analysed on a BD FACSVerse™ flow cytometer (BD Biosciences, Sparks, USA), and FlowJo software (version 7.6.5) was used to analyse the flow cytometry data.

### Elisa

Plasma IL-21 and IL-6 cytokine concentrations were assessed by ELISA (BioLegend, San Diego, CA) according to the manufacturer’s protocols. MeV-specific IgM and MeV-specific IgG antibodies in plasma samples collected within 4 days of rash onset were detected with a commercially available ELISA (Haitai Biotechnology, Zhuhai, China) according to the manufacturer’s protocol [20].

### Quantitative real-time PCR

Total RNA was extracted from the PBMCs of each patient and HC, and a reverse transcription reagent kit (Takara, Dalian, China) was used to synthesize cDNA. Quantitative real-time polymerase chain reaction (PCR) was performed to test the expression levels of target genes in triplicate with Takara SYBR Supermix (Takara, Dalian, China) as previously described [36]. The sequences of the primers used were as follows: IL-21: forward, 5′-CACAGACTAACATGCCCTTCAT-3′, and reverse, 5′-GAATCTTCACTTCCGTGTGTTCT-3′;

IL-6: forward, 5′-AGACAGCCACTCACCTCTTCAG-3′, and reverse, 5′-TTCTGCCAGTGCCTCTTTGCTG-3′;

Bcl-6: forward, 5′-CATGCAGAGATGTGCCTCCACA-3′, and reverse, 5′-TCAGAGAAGCGGCAGTCACACT-3′; and glyceraldehyde 3-phosphate dehydrogenase (GAPDH): forward, 5′-GTCTCCTCTGACTTCAACAGCG-3′; and reverse, 5′-ACCACCCTGTTGCTGTAGCCAA-3′. GAPDH was used as an internal control.

### Statistical analysis

Statistical significance was determined by the Mann-Whitney *U*-test or one-way ANOVA. Spearman correlation coefficients were used to analyse correlations between variables. All analyses were performed with GraphPad Prism 8 software (GraphPad Software, Inc., CA). All *p* values< 0.05 were considered statistically significant.

## Supplementary information

**Additional file 1: Figure S1.** Correlation of plasma MeV-specific IgG *OD* values and Tfh cells in MeV-infected patients. **a** Relationship of plasma MeV-specific IgG *OD* values and the percentage of Tfh cells; **b** Relationship of plasma MeV-specific IgG *OD* values and the percentage of ICOS^high^ Tfh cells; **c** Relationship of plasma MeV-specific IgG *OD* values and the percentage of PD-1^high^ Tfh cells. **Figure S2.** Correlation of plasma MeV-specific NAb titres and Tfh cells in MeV-infected patients. **a** MeV-specific NAb titres in Shanghai-191 vaccine strain- and wild-type strain-infected patients; **b**, **c**, **d** Relationships of plasma NAb titres with the percentages of Tfh, ICOS^high^ Tfh and PD-1^high^ Tfh cells; **e**, **f**, **g** Relationships of plasma NAb titres with the percentages of Tfh, ICOS^high^ Tfh and PD-1^high^ Tfh cells.

## Data Availability

The datasets used and/or analysed during the current study are available from the corresponding author on reasonable request.

## Abbreviations

PBMCs: Peripheral blood mononuclear cells
MeV: Measles virus
Tfh: Follicular helper T cells
IL-6: Interleukin-6
IL-21: Interleukin-21
Bcl-6: B-cell lymphoma-6
GC: Germinal centre
CXCR5: Chemokine (CXC motif) receptor 5
PD-1: Programmed death-1
ICOS: Inducible costimulator
ELISA: Enzyme-linked immunosorbent assay
HC: Healthy control
PCR: Polymerase chain reaction
GAPDH: Glyceraldehyde 3-phosphate dehydrogenase
LCMV: Lymphocytic choriomeningitis virus
HIV: Human immunodeficiency virus
SIV: Simian immunodeficiency virus
