# Supplementary material for: Elevated expansion of follicular helper T cells in peripheral blood from children with acute measles infection
Source: BMC Immunol. 2020 Sep 1;21:49. doi: 10.1186/s12865-020-00379-4 (PMC7466526; doi:10.1186/s12865-020-00379-4)
Supplement: Supplementary file 1 — Additional file 1: Figure S1. Correlation of plasma MeV-specific IgG OD values and Tfh cells in MeV-infected patients. a Relationship of plasma MeV-specific IgG OD values and the percentage of Tfh cells; b Relationship of plasma MeV-specific IgG OD values and the percentage of ICOShigh Tfh cells; c Relationship of plasma MeV-specific IgG OD values and the percentage of PD-1high Tfh cells. Figure S2. Correlation of plasma MeV-specific NAb titres and Tfh cells in MeV-infected patients. a MeV-specific NAb titres in Shanghai-191 vaccine strain- and wild-type strain-infected patients; b, c, d Relationships of plasma NAb titres with the percentages of Tfh, ICOShigh Tfh and PD-1high Tfh cells; e, f, g Relationships of plasma NAb titres with the percentages of Tfh, ICOShigh Tfh and PD-1high Tfh cells. [file 12865_2020_379_MOESM1_ESM.docx]

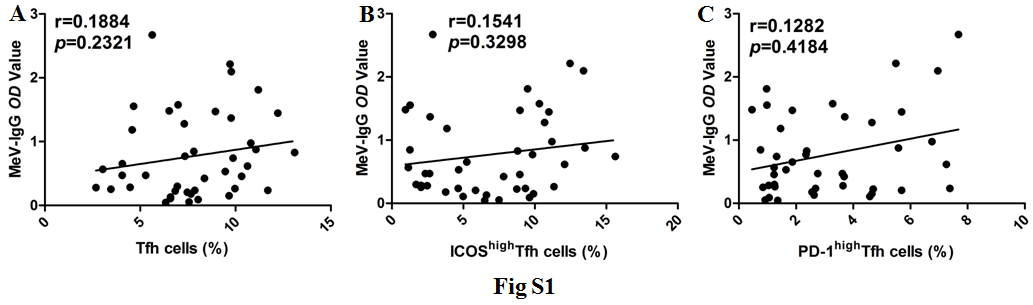


**Fig. S1** Correlation of plasma MeV-specific IgG *OD* values and Tfh cells in MeV infected cases. **a** Relationship of plasma MeV-specific IgG *OD* values and the percentage of Tfh cells; **b** Relationship of plasma MeV-specific IgG *OD* values and the percentage of ICOS^high^Tfh cells; **c** Relationship of plasma MeV-specific IgG *OD* values and the percentage of PD-1^high^Tfh cells.


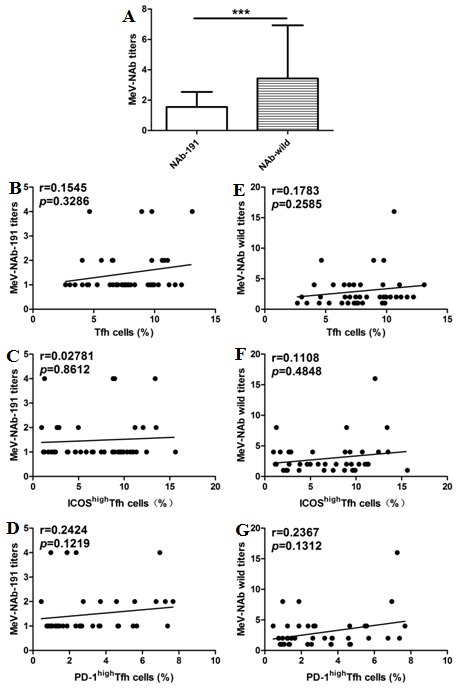


**Fig. S2** Correlation of plasma MeV-specific NAb titers and Tfh cells in MeV infected cases. **a** MeV-specific NAb titers in Shanghai-191 vaccine strain and wild-type strain; **b-d** Relationship of plasma NAb titers and the percentage of Tfh, ICOS^high^Tfh and PD-1^high^Tfh cells; **e-g** Relationship of plasma NAb titers and the percentage of Tfh, ICOS^high^Tfh and PD-1^high^Tfh cells.
